# Impact of Phosphorus Fertilization on Tomato Growth and Arbuscular Mycorrhizal Fungal Communities

**DOI:** 10.3390/microorganisms8020178

**Published:** 2020-01-25

**Authors:** Masao Higo, Mirai Azuma, Yusuke Kamiyoshihara, Akari Kanda, Yuya Tatewaki, Katsunori Isobe

**Affiliations:** Department of Agricultural Bioscience, College of Bioresource Sciences, Nihon University, 1866 Kameino, Fujisawa, Kanagawa 252-0880, Japan; azuma.mirai@nihon-u.ac.jp (M.A.); kamiyoshihara.yuusuke@nihon-u.ac.jp (Y.K.); akari.appletea@gmail.co (A.K.); bryu18009@g.nihon-u.ac.jp (Y.T.); isobe.katsunori@nihon-u.ac.jp (K.I.)

**Keywords:** amplicon sequencing, arbuscular mycorrhizal fungi, community structure, Illumina Miseq, phosphorus, tomato

## Abstract

Understanding the impact of phosphorus (P) addition on arbuscular mycorrhizal fungi (AMF) is crucial to understanding tomato (*Solanum lycopersicum* L.) P nutrition. However, it remains unknown how P fertilization is associated with the structure of AMF communities on tomato plants. Thus, we investigated whether levels of P fertilizer interacted with the colonization and structure of AMF in tomato roots in a field trial. In this study, we established three different amounts of P fertilizer treatments (0 kg ha^−1^, 50 kg ha^−1^, and 100 kg ha^−1^). We investigated AMF root colonization and community structure, as well as plant growth in tomatoes at seven weeks following transplantation. The structure of the AMF communities in the roots of tomato were determined by MiSeq amplicon sequencing. As expected, P fertilizer input enhanced the P uptake and plant biomass. In contrast, the P fertilizer level did not affect the AMF root colonization and diversity or the structure of the AMF communities in the tomato. However, we found a negative correlation between AMF colonization and richness in the roots of the tomato plants. Therefore, we need to investigate whether and how AMF communities and P fertilization develop more effective P management for tomato plants.

## 1. Introduction

Phosphorus (P) is a critical macronutrient that plays an important role in plant growth and plant metabolism. Soil P usually limits plant growth due to its poor mobility. Currently, overcoming P deficiency and increasing soil P availability is a necessary issue for achieving high crop productivity, highlighting the need to apply large amounts of inorganic P fertilizers in agricultural ecosystems [1,2,3,4]. Conversely, P has accumulated in agricultural soils due to the application of chemical fertilizers to mitigate the high P fixation capacity of acidic soils in Japan [2,5]. Thus, different P management practices are needed to develop a new technique for P fertilization to increase the P efficiency in crops [2,5].

Tomato (*Solanum lycopersicum* L.) is well known as a food that can improve human health [6,7,8]. For example, there is epidemiological evidence that tomato consumption is related to a decrease in the risk of cardiovascular diseases and cancer because the tomato plant contains high concentrations of antioxidant molecules, including carotenoids and lycopene [9]. The taste and flavor of tomatoes and their antioxidant content differ according to growing conditions, cultivation techniques, cultivars, and cultivation time [10,11,12,13]. Soil P is a well-known key nutrient for tomato production and other crops, such as maize [14] and soybean [15], and maintaining adequate levels of P in the soil is important for maximizing tomato growth and development [16,17,18]. However, the effect of different P fertilizer amounts on the growth performance of tomato plants remains unknown. Therefore, it will be useful to investigate how and whether P fertilizer levels affect the growth performance of tomato plants.

Arbuscular mycorrhizal fungi (AMF) belong to the subphylum Glomeromycotina [19,20] and form a symbiotic relationship with the majority of agricultural crops [21]. AMF improve the P uptake and growth of plants by facilitating nutrient uptake from the soil via an extensive extraradical mycelium [22,23]. The P uptake rate and efficiency can vary among the AMF taxa [24]. AMF have the ability to produce extracellular enzymes to hydrolyze organic P to enhance P uptake [25]. AMF can also mitigate the stress on plants grown in acidic soils and immobilize Al^3+^ [26]; they can also potentially improve P nutrition in host plants. Moreover, AMF are a potential solution to enhance nutrient values in crops [27]. In addition, appropriate agricultural management can improve the nutritional functions of AMF [28]. A higher AMF species richness can promote plant P nutrition [15,29,30]. In general, high levels of P fertilizer input have been shown to decrease colonization by AMF [31], as well as the abundance and richness of AMF communities in the roots and soil [32,33]. For example, high amounts of P can decrease the diversity of and change AMF communities, but moderate P fertilizer applications can enhance the diversity of AMF communities [15,34]. As mentioned above, previous reports on the impacts of P fertilization on AMF communities are contradictory. The influence of P fertilization on AMF communities can also be limited by agricultural settings. Understanding these settings is imperative to understanding how P fertilizer management is related to the nutritional benefits of AMF and tomato growth. Additionally, the effectiveness of AMF can be limited by environmental conditions, including soil P level [35,36], and our knowledge of the interactions among AMF communities in tomato roots in response to P fertilization remains unclear. Thus, how these interactions with P fertilization shape the AMF community is not fully understood.

A clear understanding of how P fertilizer managements affects AMF and tomato plants in andosols in Japan is lacking. P fertilization may shift AMF communities toward an unfavorable condition, as tomato plants can be less dependent on AMF for P uptake; on the other hand, AMF species may be favored under P-fertilized conditions. Therefore, we hypothesized that P fertilizer management in tomato cultivation would induce a shift in the AMF community and that this change would be associated with the P uptake and growth of the tomatoes in a field experiment. Thus, we conducted our field investigation with two purposes: (1) to understand whether P fertilizer management increases tomato growth performance and (2) to determine whether and how the AMF communities in tomato roots are influenced by P fertilizer management.

## 2. Materials and Methods

### 2.1. Experimental Design

We performed a field experiment at Nihon University in Kanagawa, Japan (35.380069, 139.465313), to investigate the influence of P fertilizer levels on soil biochemical properties and tomato growth. The field soil’s classification is an allophonic andosol (a volcanic ash soil). In this study, two levels of P fertilizer (triple superphosphate) treatments, including 50 kg ha^−1^ (low-P) and 100 kg ha^−1^ (high-P), were established for tomato cultivation (*Solanum lycopersicum* L., cv: Rio Grande). We also established a control treatment (zero-P) where no P fertilizer was applied. A randomized complete block design was arranged with three replicate plots per treatment. The N and K_2_O application rates in tomato cultivation were both 100 kg ha^−1^. Ammonium sulfate and potassium chloride were used for fertilization in this study. All fertilizers were manually broadcasted into each plot and incorporated into the soil on 24 April 2018. Tomato plantlets were prepared on sterilized soil in a greenhouse and were maintained in a nursery for 50 days. They received a Hoagland solution once a week. Tomato plantlets were transplanted to the field at a spacing of 75 cm × 40 cm within a 3 m × 3 m plot on 26 April 2018 when they had four to five true leaves. We watered the tomato plants using a sprinkler.

### 2.2. Soil Sampling and Measurement of Soil Biochemical Properties before Transplanting Tomatoes

On 24 April 2018, we randomly collected soil samples from ten points (with a 4 cm diameter and a 0–20 cm depth) using a core sampler (DIK-102A, Daiki Rika Co., Ltd., Saitama, Japan) in each plot and combined the samples into one composite material after applying the fertilizers. Soil pH (a soil/water ratio of 1:2.5 w/v) and electrical conductivity (EC) (soil/water ratio of 1:5 w/v) were determined with a digital pH and conductivity meter (HI 9811, HANNA). Soil available P (extractable P) was extracted according to Bray and Kurtz [37] and measured by the molybdenum blue method at 710 nm using a UV-1700 Spectrophotometer (Shimadzu Co. Ltd., Kyoto, Japan). The activities of acid phosphatase (ACP), alkaline phosphatase (ALP), and β-glucosidase in soil were measured according to Ishii and Hayano [38] and Hayano [39].

### 2.3. Estimation of Tomato Growth Parameters

The shoots of six tomato plants per plot were cut close to the ground at seven weeks following transplantation (flowering stage) on 31 May 2018. The shoot biomass, plant length, soil–plant analysis development (SPAD) value, and leaf area were measured in all plots. The SPAD value was measured using a SPAD-502 Plus (Minolta Camera Co. Ltd., Osaka, Japan). The leaf area of the tomato plants was determined using a leaf area meter LI-3100C (Li-Cor, Lincoln, NE, USA). The shoot biomass for the tomato plants was determined after the samples were oven-dried at 80 °C for 48 h. For the analysis of shoot P concentration and P uptake, six dried plant samples per plot were homogenized using a commercial homogenizer and pooled into one composite sample. The shoot P concentration was determined using the vanadium–molybdenum yellow colorimetric method after the digestion of plant tissue using 60% perchloric acid [40]. After digestion of the shoot tissues, we measured the plant’s P concentration using the vanadium–molybdenum yellow colorimetric method at 410 nm with a UV-1700 Spectrophotometer.

### 2.4. Root Sampling and Staining

Tomato root samples were taken from six plants of each plot seven weeks following transplantation (flowering stage) on 31 May 2018. The roots were stored at −80 °C to estimate colonization by AMF and DNA extraction. The fresh roots were stained with a 3,3′-diaminobenzidine (DAB) solution [41]. The colonization by AMF in the tomato plants was estimated according to the method of Giovannetti and Mosse [42]. The colonization by AMF was examined from a 200-point gridline intersection of the root using a stereomicroscope (SZX12, Olympus, Tokyo, Japan).

### 2.5. Extraction of Genomic DNA and Polymerase Chain Reaction (PCR)

From a composite sample taken from every plot, we collected 100 mg of fresh roots from several fine roots at various places on the plant root system. Then, genomic DNA was obtained from 100 mg of fresh roots per plot using the NucleoSpin^®^ Plant II kit (Macherey–Nagel, Duren, Germany). We extracted and prepared a total of 9 genomic DNA samples in this study. We stored the extracted DNA solutions at −30 °C. After DNA extraction, we amplified the partial sequences in the 18S rDNA based on the PCR procedure [43]. A primer pair of NS31 [44] and AM1 [45] was selected for the first step of the PCR to amplify the partial sequences of the 18S rDNA gene for Glomeromycotina [19,20]. To reduce potential errors and biases in the PCR process, each sample was prepared and amplified in triplicate [46]. The genomic DNA from three subsamples per replicate were amplified for a total of 27 PCR reactions (because we used Illumina amplicon sequencing to evaluate the effects of three P treatments with each reaction replicated three times). Each PCR was carried out in 10 μL containing 0.4 μM of forward and reverse primers, 2 × Platinum™ Green PCR Master Mix (Thermo Fisher Scientific Inc., Waltham, MA, USA), and 1 µL of template. The first-step PCR conditions comprised an initial treatment at 94 °C for 2 min; 30 cycles at 94 °C for 30 s, at 56 °C for 30 s, and at 72 °C for 30 s, and then a final extension at 72 °C for 5 min. The first-step PCR products were diluted 10-fold and used as templates for the second PCR using the primer pair of AMV4.5NF/AMDGR [47]. Each second-step PCR was performed in 20 μL containing 0.3 μM of forward and reverse primers, 2 × Platinum TM Green PCR Master Mix, and 2 µL of the template. The second-step PCR protocol was composed of an initial treatment at 98 °C for 2 min; 40 cycles of treatments at 98 °C for 10 s, at 60 °C for 10 s, and at 72 °C for 15 s, and a final extension at 72 °C for 10 min. The PCR products (300 bp) were analyzed on a 1% agarose gel using gel electrophoresis and visualized using Atlas ClearSight DNA Stain (BioAtlas, Tartu, Estonia) on a UV transilluminator (Image Saver AE-6905C, ATTO).

### 2.6. Amplicon Sequencing for AMF Communities in Roots

To characterize the AMF community in the tomato roots, we performed high-throughput sequencing of amplicons as described in Higo et al. [15,43,48]. Briefly, three PCR products per plot were combined together (from 27 samples to 9 samples) to prepare 9 libraries. These libraries were purified by NucleoSpin Gel and a PCR Clean-up kit (Macherey–Nagel) to limit potential PCR biases and quantified by UV spectrophotometry (DS-11 NanoPad, DeNovix Inc., Wilmington, DE, USA). We normalized the purified PCR product before amplicon sequencing. We pair-end (PE; 2 × 300 bp) sequenced the purified products to form consensus sequences on Illumina MiSeq equipment (V3 MiSeq Reagent Kit). After PE sequencing, we performed sequence read processing by Quantitative Insights Into Microbial Ecology (QIIME) 2.0 [49].

The analysis of sequences after running the Illumina MiSeq amplicon sequencing was performed according to Higo et al. [43]. All sample data after the MiSeq amplicon sequencing were filtered using the FASTX-Toolkit. Briefly, using the FASTX-Toolkit’s fastq_barcode_spliltter, only read sequences matched precisely with the used primers were extracted. We removed the forward and reverse primers, the barcode and 70 bp from the 3′ end of and then assembled the PE sequences. Next, we denoised the PE sequences using DADA2 in QIIME 2.0 [49,50]. We also used DADA2 to remove chimeric sequences along with sequence singletons and to denoise sequences into amplicon sequence variants (ASVs). The raw data of the sequences are available in the Sequence Read Archive (SRA) of the DNA Data Bank of Japan (DDBJ) (Bio Project Accession: PRJDB7942). Rarefaction analysis of the lowest reads (21,357 sequences) per sample among the treatments was carried out using the “*rarefy*” function in the R package vegan in R 3.5.2 [51]. After rarefaction analysis, we removed rare ASVs (i.e., ASVs with fewer than 10 sequences within a sample according to the process of Lindahl et al. [52] and Oliver et al. [53]).

### 2.7. Statistical Analysis

Significant differences among P fertilizer levels in each variable (soil biochemical properties, plant growth parameters, and AMF diversity) were determined by a Tukey’s test using the R package emmeans [54]. For the community analysis, we performed resampling to the lowest ASV abundance to assess differences between treatments regardless of the sequencing depth for non-metric multidimensional scaling (NMDS) and a permutational multivariate analysis of variance (PERMANOVA). In addition, we assessed Hill numbers, such as ASV richness, Shannon diversity (the exponential of entropy), and Simpson diversity (the inverse of the Simpson index), to determine the structure of the AMF communities in each P fertilizer treatment using the R package vegan. We also calculated the mean nearest taxon distance (MNTD) as the phylogenetic diversity for the α diversity analysis using the R package picante [55]. Shared ASVs between the two P fertilizer treatments and the control group were analyzed with a Venn diagram (R package eulerr version 5.1.0) [56].

The variations in the structures of AMF communities among the P fertilizer levels were also examined by the NMDS using the “*metaMDS*” function in the vegan package. To investigate whether P fertilizer levels significantly changed the structure of the AMF communities in tomato roots, PERMANOVA was carried out with 999 permutations by using the “*adonis*” function in the R package vegan. The Bray–Curtis distance of the ASV or AMF genus matrix [57] was selected in PERMANOVA. Moreover, we investigated the nestedness of the AMF communities using R package bipartite version 2.13 [58,59,60]. We also calculated the nestedness based on overlap and decreasing fill (NODF) [61] on a scale from 0 to 1. The significance of the network property for nestedness was estimated by the quantitative r2dtable method in the R package vegan. Significant positive z-scores indicated that there is a nested structure in the matrix, and significant negative z-scores indicated antinestedness. For the nestedness analysis, the ASVs with low abundance (less than 0.5%) were removed from the dataset.

## 3. Results

### 3.1. Influence of P Fertilizer Level on Soil Biochemical Properties

We found that there were no significant differences in the variables except for EC, NO_3_-N, and soil available P content between the different amounts of P fertilizer treatments before tomato cultivation (Table 1). The soil available P in the zero-P (0 kg ha^−1^) treatment was significantly lower than the low-P (50 kg ha^−1^) and high-P (100 kg ha^−1^) treatments. The EC in the zero-P treatment was significantly lower compared to the high-P treatments. NO_3_-N content was significantly different among the P treatments. No significant differences in the soil pH among the P fertilizer treatments were found. The activities of ACP and ALP were not also affected by the P fertilizer level.

### 3.2. Influence of P Fertilizer Level on Plant Growth, P Uptake and AMF Colonization of Tomato Plants

The plant length and leaf area of the tomatoes at seven weeks following transplantation significantly varied among the P fertilizer treatments (Table 2). The plant lengths under the high-P treatment were significantly higher than those under the zero-P and low-P treatments. Additionally, one tomato plant’s leaf area in the zero-P treatment was significantly lower than that in the low-P and high-P treatments. On the other hand, the SPAD value in the tomato plants did not differ between all P fertilizer treatments (Table 2). Moreover, the shoot biomass in the tomato at seven weeks following transplantation significantly varied among the P fertilizer treatments (Table 3). The shoot biomass under the high-P treatment was significantly higher than that in the zero-P and low-P treatments. P fertilizer input also had a significant effect on the plant P concentration and uptake when the amount of P fertilizer input was increased (Table 3). The plant P concentration and uptake under high-P treatment were significantly greater than those under the zero-P and low-P treatments.

**Figure 1:**
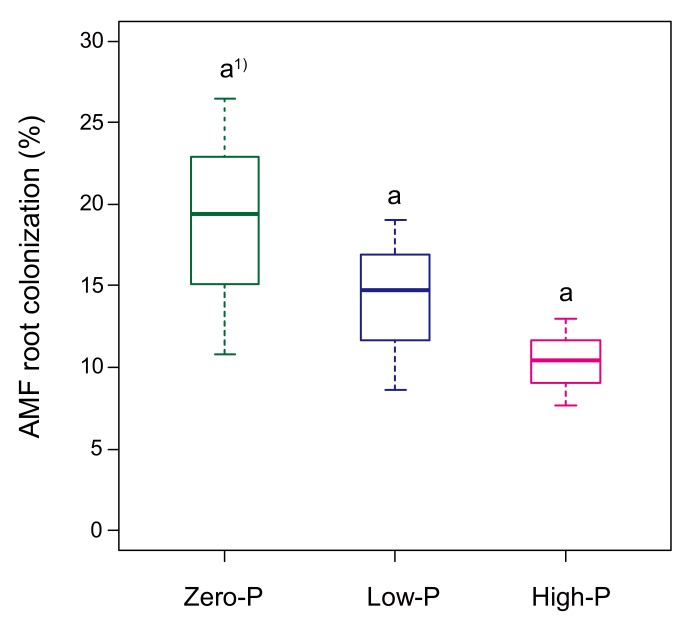
Impact of the different amount of P fertilizer on the arbuscular mycorrhizal fungi (AMF) in the roots of tomato plants at seven weeks following transplantation. Horizontal lines show the median; box margins ± standard error and vertical lines show the minimum and maximum values of the treatments. ^1)^ Different letters show a significant difference according to a Tukey’s test among the P fertilizer levels (*p* < 0.05).

In our study, the colonization by AMF under the zero-P treatment (18.9%) tended to be higher than that in the low-P (14.2%) and high-P treatments (10.4%) (Figure 1), although there were no significant differences in the colonization by AMF for all P fertilizer levels.

### 3.3. General Sequencing Information and Molecular Diversity of AMF Communities

**Figure 2:**
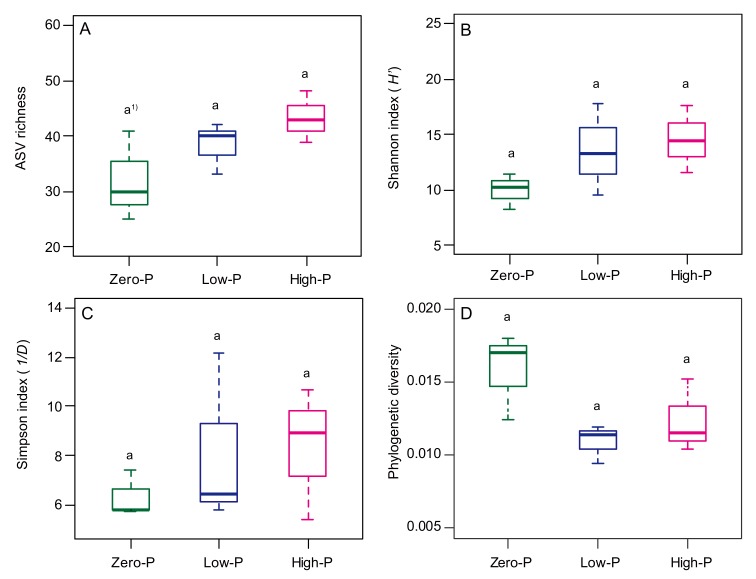
Impact of the different amounts of P fertilizer on (**A**) amplicon sequence variant (ASV) richness, (**B**) Shannon index (the exponential of entropy), (**C**) Simpson index (inverse of the Simpson index), and (**D**) mean nearest taxon distance (NTI) of the AMF community structure in the roots of tomato plants at seven weeks following transplantation. Horizontal lines show the median; box margins ± standard error and vertical lines show the minimum and maximum values of the treatments. ^1)^ Different letters show a significant difference according to Tukey’s test among the P fertilizer levels (*p* < 0.05).

A total of 353,874 paired-end sequences were derived from the nine libraries. Of these, 245,706 sequences corresponded to Glomeromycotina. We found a total of 117 amplicon sequence variants (ASVs) belonging to Glomeromycotina in the roots (Table S1). The samples from roots in the tomato under all P fertilizer treatments showed similar diversity in their ASV richness, Shannon index, and Simpson index (Figure 2A–C). Moreover, no significant differences among the P fertilizer treatments were found. The phylogenetic diversity of AMF communities was not also influenced by P fertilization (Figure 2D).

### 3.4. Influence of P Fertilizer Level on the AMF Communities in Tomato

**Figure 3:**
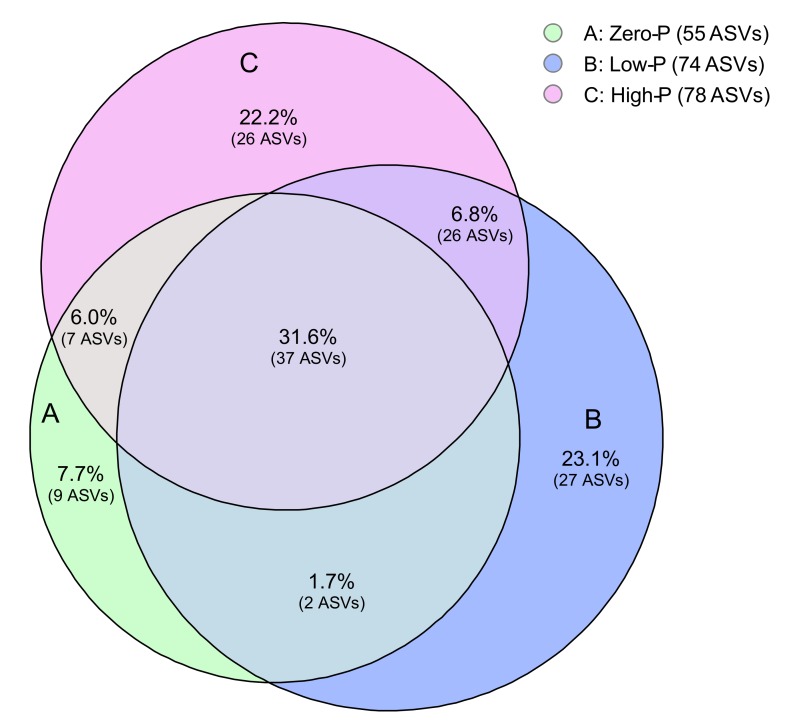
Impact of the different amounts of phosphorus fertilizer on the overlap of amplicon sequence variants (ASVs) in the roots of tomato plants at seven weeks following transplantation. Numbers inside the Venn diagram indicate specific and shared ASVs.

**Figure 4:**
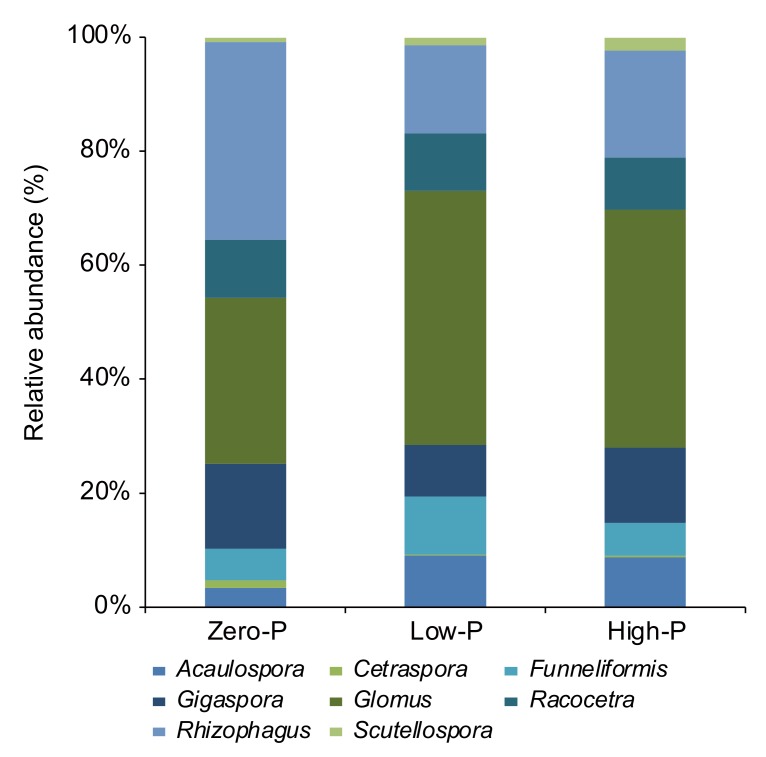
Impact of the different amounts of phosphorus fertilizer on the genus-based arbuscular mycorrhizal fungal communities in the roots of tomato plants at seven weeks following transplantation.

We found that 31.6% of ASVs were common under all P fertilizer levels (Figure 3). The ASVs that occurred specifically in only the zero-P, low-P, and high-P treatments comprised 7.7%, 23.1%, and 22.2%, respectively. Additionally, we found that the relative abundance of AMF ASVs in tomato roots tended to be similar among P fertilization groups (Figure 4). Glomeraceae was predominant and observed at a much higher frequency (68.7%) than other AMF groups in roots (Figure 4). The relative abundance of Gigasporaceae and Acaulosporaceae was 24.2% and 7.1%, respectively. In addition, the relative abundance of *Funneliformis*, *Glomus*, and *Rhizophagus* was 7.2%, 38.5%, and 24.2%, respectively. The relative abundance of *Cetraspora*, *Gigaspora*, *Racocetra*, and *Scutellospora* was 0.6%, 12.4%, 9.7%, and 1.5%, respectively.

The relative abundance of *Rhizophagus* also tended to decrease as a result of P fertilization. Moreover, the related ASVs of *Rhizophagus clarus* (ASV194; Accession No. KP144311) were observed at a much higher frequency in roots (23.8%) (Figure S2 and Table S1). In turn, the relative abundance of uncultured *Glomus* (ASV973; Accession No. AB326021), *Gigaspora margarita* (ASV638; Accession No. KP677606), uncultured Glomeromycotina (ASV845; Accession No. KX108189), and *Funneliformis mosseae* (ASV908; Accession No. AY635833) was 16.4%, 6.5%, 5.2%, and 6.2%, respectively.

**Figure 5:**
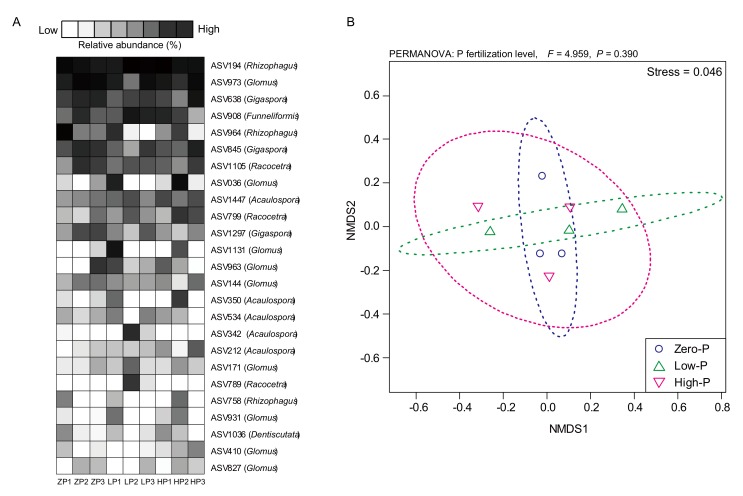
Impact of the different amounts of P fertilizer on the communities of arbuscular mycorrhizal fungi (AMF) colonizing tomato roots at seven weeks following transplantation. (**A**) The nestedness of dominant AMF amplicon sequence variants (ASVs) in the P fertilizer treatments. The ASVs were ordered by decreasing ASV abundance (top to bottom), and the name of the AMF genus is indicated on the right of the matrix. ZP: zero-P, LP: low-P, and HP: high-P treatments. Significance of the nestedness structure (zero-P: z-score = −1.756 *p*-value = 0.079, low-P: z-score = 1.602, *p*-value = 0.109, high-P: *z*-score = −2.834, *p*-value = 0.005 **). (**B**) Non-metric multidimensional scaling (NMDS) illustrating the relationship among the structures of the AMF communities and P fertilizer treatments in tomato plants. Ellipses represent confidence intervals at 95%.

Additionally, we used a nestedness analysis to investigate the patterns of species occurrence among a set of treatments (e.g., P fertilizer levels) and the distribution patterns of interacting AMF taxa within ecological networks. The nestedness structure analysis showed that the AMF communities in tomato roots were randomly distributed among P fertilizer levels (Figure 5A). Furthermore, we used NMDS to determine the differences in the structures of AMF communities in the roots of tomato among P fertilizer treatments (Figure 5B). The results of the NMDS showed that P fertilizer levels did not affect the shift in the structure of AMF communities (Figure 5B). PERMANOVA was also performed to investigate the significant differences in the structure of AMF communities in tomato among different P fertilizer levels (P fertilizer levels; *F* = 4.959, *p* = 0.390).

**Figure 6:**
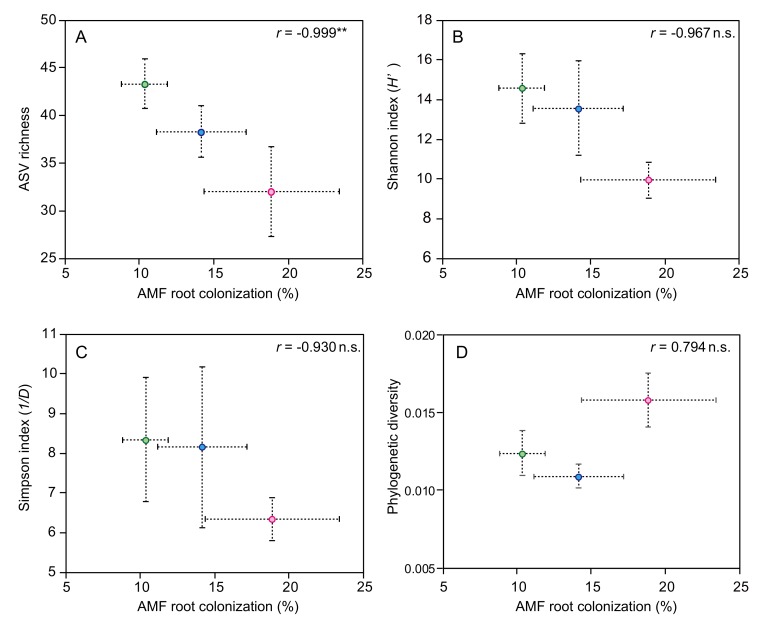
Correlation between AMF root colonization and AMF diversity variables in tomato roots at seven weeks following transplantation. (**A**) AMF colonization vs. ASV richness, (**B**) AMF colonization vs. Shannon index (the exponential of entropy), (**C**) AMF colonization vs. Simpson index (inverse of the Simpson index), (**D**) AMF colonization vs. mean nearest taxon distance (NTI). *r* shows the Pearson correlation coefficient. n.s.: not significant, and ** indicates a significant difference in the Pearson correlation coefficient between the two variables at a 0.1% level.

We also found that the relationship between ASV richness and AMF root colonization (*r* = −0.999) was negatively and significantly correlated to P fertilizer levels (Figure 6A). The relationships between the Shannon and Simpson indices and AMF root colonization (*r* = −0.967, *r* = −0.930) tended to be negatively correlated to P fertilizer levels (Figure 6B,C). In contrast, the relationships between the phylogenetic diversity and AMF colonization (*r* = 0.794) were not linearly correlated to the P fertilizer levels (Figure 6D).

## 4. Discussion

### 4.1. Influence of P Fertilizer Level on Tomato Growth

In this study, the growth of tomato plants significantly improved by increasing the P fertilizer level (Table 2), as in an earlier study [62,63]. Crop responses to P fertilization differed with P fertilizer levels and the availability of other nutrients [63,64,65,66,67]. In addition, the P concentration in plant tissues and their growth and nutrient acquisition can be mediated by the complex interactions between climate, soil, and agricultural management conditions [68]. Among the complex interactions of soil and agricultural management conditions, soil pH plays an important role in the conversion of organic P into soluble P [69,70]. Our research field’s soil was classified as volcanic acid soil with a high P-fixation capacity. Thus, we need to consider the interactions between soil pH and the plant availability of P to improve tomato growth and P uptake. Moreover, Maherali and Klironomos [71] indicated that the high level of extraradical hyphal growth in Gigasporaceae compared to other AMF families, such as Glomeraceae and Acaulosporaceae, is correlated to enhanced P concentrations in the shoots of *Plantago lanceolata*. Thus, the detected AMF ASVs in the Gigasporaceae (24.2% relative abundance, including *Cetraspora*, *Gigaspora*, *Racocetra*, and *Scutellospora*) used in this study may be related to the improvement of tomato growth and fruit parameters compared to the other AMF ASVs. However, there is no evidence that these detected AMF taxa contributed to the growth and P uptake of tomato plants in our study. Thus, future approaches, such as inoculation experiments, using each AMF taxon will be needed to improve tomato growth performance.

### 4.2. Effect of P Fertilizer Level on the AMF Communities in Tomato Roots

Our findings showed that Glomeraceae, including *Glomus*, *Rhizophagus*, and *Funneliformis*, were the main family in tomato roots, but Gigasporaceae, including *Cetraspora*, *Gigaspora*, *Racocetra*, and *Scutellospora*, and Acaulosporaceae were also detected in tomato roots (Figure 4 and Figure S2). Previous studies using the Illumina MiSeq Platform have shown that Glomeraceae are predominant in roots and soils [43,48,72,73,74]. Oehl et al. [75], as the result of a pot experiment, reported that Glomeraceae colonize plant roots through pieces of hyphae or colonized roots, thereby quickly constructing hyphal anastomosis [76] and possessing the ability to restore hyphal networks after mechanical disruptions. However, another family, Gigasporaceae, spreads through spores from an intact mycelium [77]. Thus, the fungal characteristics of Glomeraceae facilitate their spread and propagation in agroecosystems, and the frequency of this phenomenon could be the outcome of their adjustment to agricultural conditions.

In addition, a higher diversity of AMF communities in agricultural settings has been indicated to possess abilities to improve plant growth performance [24]. Many studies have reported that fertilization remarkably reduces AMF diversity [78] and that high levels of P application decrease the diversity of AMF communities [32,79]. Our results indicated no effect on the AMF communities in roots (Figure 1; Figure 4, Table S2), but we found a negative correlation between AMF colonization and richness in this study (Figure 6A, Table S3). A previous finding also showed that P application had no effect on the diversity of AMF communities in soil and roots under a long-term field experiment [14], which is in partial agreement with our findings. Liu et al. [80] showed that the structures of AMF communities in maize roots are significantly influenced by growth stage (6-leaf collar, 13-leaf collar, and kernel dough stages), but not by P fertilizer levels (0 kg, 25 kg, and 100 kg P ha^−1^). Liu et al. [80] also indicated that crop phenology may be a stronger determinant than P application in shaping the AMF community structure in roots. Along with AMF communities, Johnson [81] indicated that N fertilization under low P availability may enhance the C supply in the soil. One explanation for why the AMF colonization in tomato roots was not inhibited by increasing the application of P is that AMF colonization is closely associated with the N nutrition of crops. In this experiment, N fertilizer was applied at a rate intended to induce optimal tomato growth; thus, N nutrition in tomato plants may inhibit AMF root colonization by increasing P fertilization.

Furthermore, the P fertilization management in this region may mean that certain AMF taxa are selected because they have weak responses to P fertilization. In fact, Higo et al. [15] indicated that the abundance of AMF taxa (*Claroideoglomus claroideum*, *Funneliformis mosseae*, and *Diversispora celata*) was not influenced by P fertilization. *Rhizophagus irregularis* were found in various types of fields and may have a high tolerance for environmental drivers [82]. Some of these P-unresponsive species are predominant in the same experimental fields that we used in the current study. Thus, one possible explanation for the outcome is that the hyphal elongation by AMF in the soil could not be inhibited by P fertilizer treatments. This could be one reason why P fertilizer input did not change the diversity of the AMF communities in tomato plants. Additionally, the impact of soil P on the diversity of AMF communities still remains a controversial subject, and the results could be related to P application rates [80], sampling times [83], host plant species [84,85], and agricultural management [86,87,88,89,90]. Our results were inconsistent regarding P fertilization on the diversity of AMF communities compared to previous reports. This discrepancy may be related to different factors including the rate of P fertilizer, soil biochemical properties, and climatic conditions. Thus, further studies into the relationships among AMF taxa will be needed to illustrate the interactions between P fertilization and AMF communities in tomato cultivation systems.

## 5. Conclusions

The P fertilizer level (0–100 kg ha^−1^) was not a strong driver for forming the structure of AMF communities in tomato roots. One possible reason for this may be that this is the result of a seven-week study, so the differences in the AMF communities caused by P fertilizer input remained unclear in the tomato roots of the cropping system. Another possible reason is a potential limitation of experimental design with three repetitions per treatment in the present study, which failed to support our hypothesis, as there were no differences in diversity and communities of AMF among P fertilizer treatments. We will need to conduct continuous experiments to determine how P fertilizer management can alter the diversity of AMF communities in tomatoes in a long-term field experiment. We also confirmed that AMF root colonization had a negative correlation with the richness of AMF communities in the roots of tomato. This may mean that less C investment by the plant to AMF could relate to higher P conditions, which may lead to less colonization by AMF. Thus, a high diversity of AMF could reveal parasitism by AMF and intense competition between AMF at high P conditions. However, we could not distinguish between how much P was transported via tomato plants and how much P was transported via AMF or other factors. These results will be important to understand the relationship among AMF diversity, P fertilization, and the plant P nutrition of tomatoes.

## Figures and Tables

**Table 1:** Effect of the different amounts of phosphorus (P) fertilizer on the soil biochemical properties before the cultivation of tomato plants.

**P Fertilizer Levels**	**Soil pH**			**EC**			**Available Soil P Content**		
	**(H_2_O)**			**(μS/cm)**			**(mg/kg)**		
Zero-P	5.7	(0.01) ^1^	a ^2^	53.3	(3.33)	b	17.0	(3.9)	b
Low-P	5.7	(0.03)	a	74.4	(2.22)	a	19.1	(1.6)	b
High-P	5.7	(0.07)	a	84.4	(7.29)	a	54.3	(12.9)	a
**P fertilizer levels**	**NO_3_-N Content**			**ACP Activity**			**ALP Activity**		
	**(mg/kg)**			**(mU/g)**			**(mU/g)**		
Zero-P	28.7	(0.2)	a	20.1	(0.3)	a	53.9	(2.8)	a
Low-P	25.1	(0.8)	b	27.9	(1.2)	a	61.8	(1.2)	a
High-P	22.1	(0.6)	c	34.2	(8.4)	a	59.1	(1.0)	a

^1^ Numbers show the mean values of *n* = 3 with standard errors in parentheses. ^2^ Different letters within the same column for each parameter among the P treatments indicate a significant difference according to Tukey’s test (*p* < 0.05). EC: electrical conductivity; ACP: acid phosphatase; ALP: alkaline phosphatase.

**Table 2:** Effect of the different amounts of P fertilizer on the growth parameters of tomato plants at seven weeks following transplantation.

P Fertilizer Levels	Plant Length			SPAD Value			Leaf Area		
	(cm)						(cm^2^/plant)		
Zero-P	19.5	(3.5) ^1^	b ^2^	48.9	(1.4)	a	83.5	(15.6)	b
Low-P	22.3	(2.1)	b	49.1	(0.8)	a	364.0	(85.4)	a
High-P	39.6	(3.2)	a	51.5	(0.3)	a	350.9	(24.4)	a

^1^ Numbers show the mean values of *n* = 3 with standard errors in parentheses. ^2^ Different letters within the same column for each parameter among the P treatments indicate a significant difference according to Tukey’s test (*p* < 0.05). SPAD: soil–plant analysis development.

**Table 3:** Effect of the different amount of P fertilizer on the shoot biomass and shoot P uptake at seven weeks following transplantation.

P Fertilizer Levels	Shoot Biomass			P Concentration			P Uptake		
	(g/m²)			(mg P/g)			(mg P/m²)		
Zero-P	2.9	(1.2) ^1^	b ^2^	1.6	(0.09)	b	4.6	(1.9)	b
Low-P	5.2	(1.1)	b	1.7	(0.06)	b	8.9	(2.1)	b
High-P	11.3	(1.1)	a	2.2	(0.08)	a	24.7	(3.4)	a

^1^ Numbers show the mean values of *n* = 3 with standard errors in parentheses. ^2^ Different letters within the same column for each parameter among the P treatments indicate a significant difference according to Tukey’s test (*p* < 0.05).

## Supplementary Materials

The following are available online at https://www.mdpi.com/2076-2607/8/2/178/s1.
