# Supplementary material for: Impact of Phosphorus Fertilization on Tomato Growth and Arbuscular Mycorrhizal Fungal Communities
Source: Microorganisms. 2020 Jan 25;8(2):178. doi: 10.3390/microorganisms8020178 (PMC7074694; doi:10.3390/microorganisms8020178)
Supplement: Supplementary file 1 [file microorganisms-08-00178-s001.zip › microorganisms-679552-supp final check/Figures S1 and S2.pdf]

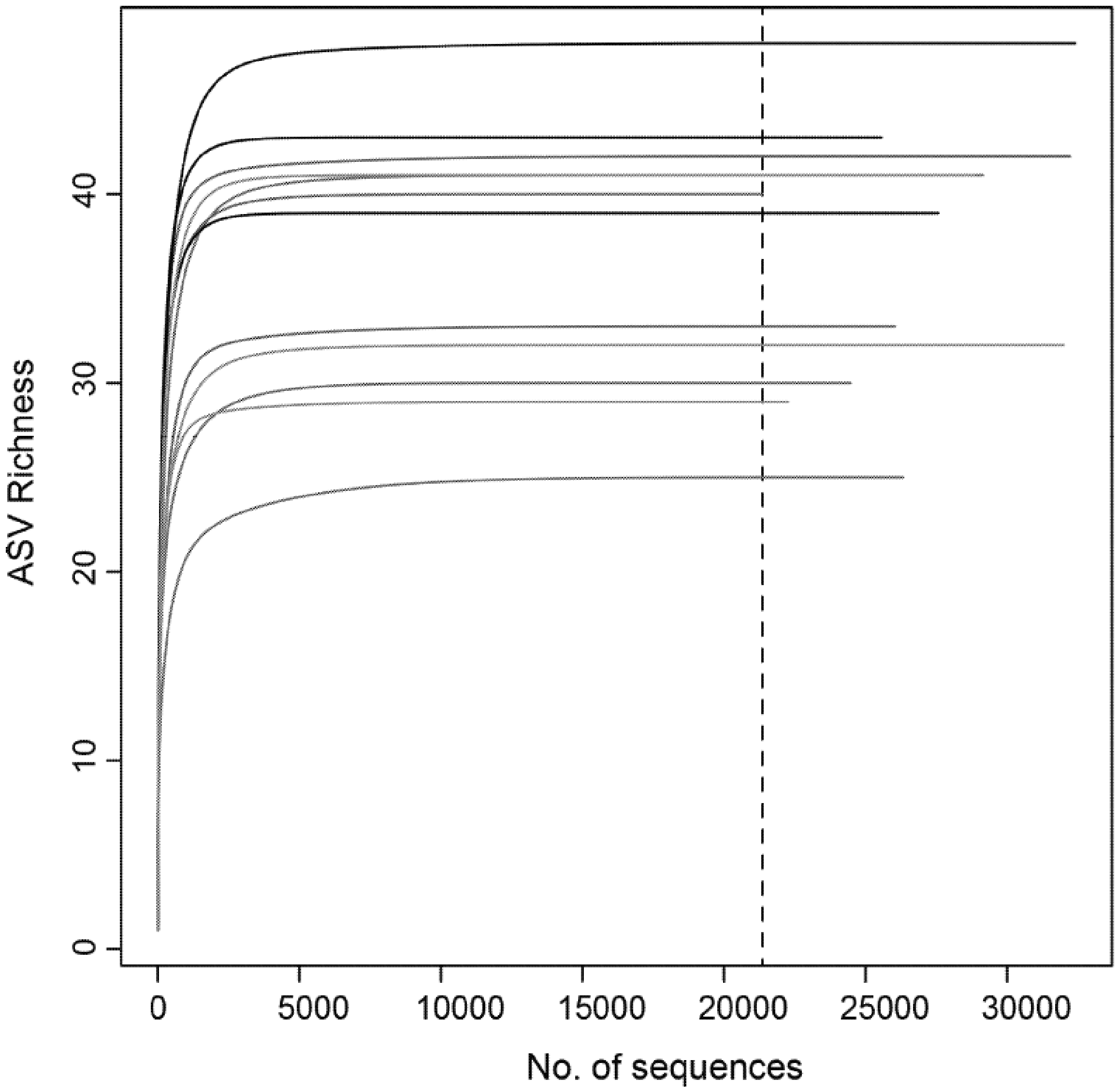

Figure S1. Rarefaction curves illustrating the sequencing depths in the roots of tomato plants. The vertical lines indicate a minimum (21,357 sequences) number of detected sequences. Zero-P: red, low-P: blue, and high-P: green.

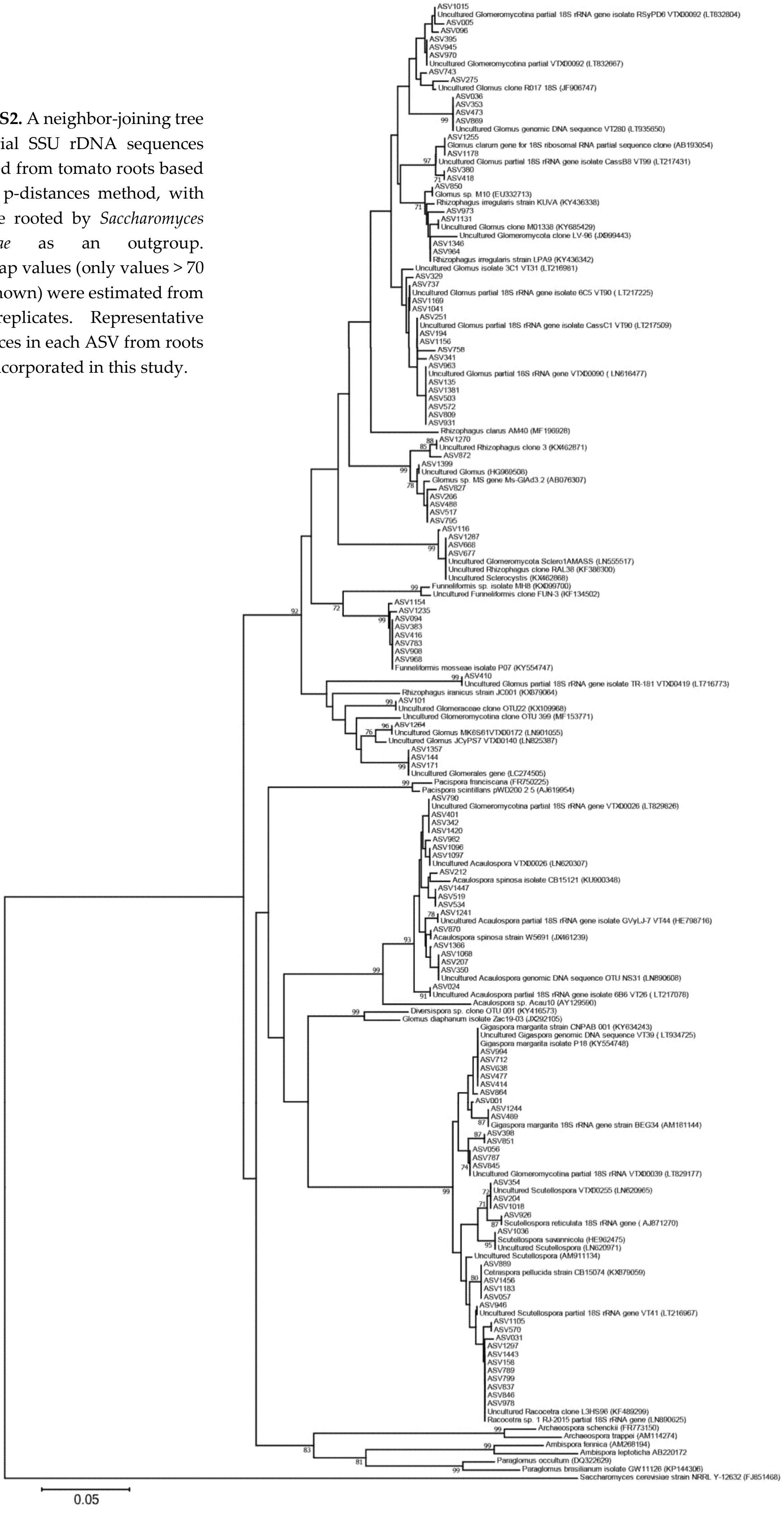

Figure S2. A neighbor-joining tree of partial SSU rDNA sequences obtained from tomato roots based on the p-distances method, with the tree rooted by *Saccharomyces cerevisiae* as an outgroup. Bootstrap values (only values > 70 were shown) were estimated from 1000 replicates. Representative sequences in each ASV from roots were incorporated in this study.
